# Muscarinic acetylcholine receptor M5 is involved in spermatogenesis through the modification of cell–cell junctions

**DOI:** 10.1530/REP-21-0079

**Published:** 2021-05-10

**Authors:** Xiao Han, Cong Zhang, Xiangping Ma, Xiaowei Yan, Bohui Xiong, Wei Shen, Shen Yin, Hongfu Zhang, Qingyuan Sun, Yong Zhao

**Affiliations:** 1 State Key Laboratory of Animal Nutrition, Institute of Animal Sciences, Chinese Academy of Agricultural Sciences, Beijing, People’s Republic of China; 2 College of Life Sciences, Qingdao Agricultural University, Qingdao, People’s Republic of China; 3 Fertility Preservation Lab, Reproductive Medicine Center, Guangdong Second Provincial General Hospital, Guangzhou, People’s Republic of China

## Abstract

Muscarinic acetylcholine receptor (mAChR) antagonists have been reported to decrease male fertility; however, the roles of mAChRs in spermatogenesis and the underlying mechanisms are not understood yet. During spermatogenesis, extensive remodeling between Sertoli cells and/or germ cells interfaces takes place to accommodate the transport of developing germ cells across the blood-testis barrier (BTB) and adluminal compartment. The cell–cell junctions play a vital role in the spermatogenesis process. This study used ICR male mice and spermatogonial cells (C18-4) and Sertoli cells (TM-4). shRNA of control or M5 gene was injected into 5-week-old ICR mice testes. Ten days post-viral grafting, mice were deeply anesthetized with pentobarbital and the testes were collected. One testicle was fresh frozen for RNA-seq analysis or Western blotting (WB). The second testicle was fixed for immunofluorescence staining (IHF). C18-4 or TM-4 cells were treated with shRNA of control or M5 gene. Then, the cells were collected for RNA-seq analysis, WB, or IHF. Knockdown of mAChR M5 disrupted mouse spermatogenesis and damaged the actin-based cytoskeleton and many types of junction proteins in both Sertoli cells and germ cells. M5 knockdown decreased Phldb2 expression in both germ cells and Sertoli cells which suggested that Phldb2 may be involved in cytoskeleton and cell–cell junction formation to regulate spermatogenesis. Our investigation has elucidated a novel role for mAChR M5 in the regulation of spermatogenesis through the interactions of Phldb2 and cell–cell junctions. M5 may be an attractive future therapeutic target in the treatment of male reproductive disorders.

## Introduction

Both the superior (from the coeliac and aortic plexuses) and inferior (from the inferior mesenteric and hypogastric plexuses) spermatic nerves innervate the testes and are involved in fine-tuning the regulation of testicular functions (Gerendai & Halász 1997, Gerendai 2004). Other studies have shown that chronic testicular denervation in mature rats leads to the disruption of male germ cell development (Chow* et al.* 2000), thus emphasizing the vital role of the nervous system in spermatogenesis. Moreover, cholinergic fibers are known to regulate steroidogenesis in the testis (Zhu* et al.*2002).

Cellular actions of the ancient signaling molecule acetylcholine (ACh) are mediated by two types of membrane receptors: nicotinic receptors and muscarinic ACh receptors (mAChRs; Wessler* et al.* 1998, Eglen 2006). These receptors are widely present in the central and peripheral nervous systems (Wessler* et al.* 1998, Eglen 2006). mAChRs are members of the G protein-coupled receptor (GPCR) family and include five subtypes (M1–M5) that are encoded by five different genes (Eglen 2006).

M1, M3, and M5 mAChRs mainly couple with Gq_/11_ to activate the phosphoinositide-specific phospholipase Cβ (PLCβ) to produce inositol 1,4,5-triphosphate (IP3) and 1,2-diacylglycerol (DAG), followed by the elevation of intracellular Ca^2+^ and the activity of protein kinase C (PKC). M2 and M4 mainly couple with Gi/o to produce protein-dependent signaling. M1–M4 mAChRs subtypes have been studied for a long time, however, M5 is the least investigated mAChR subtype and was the last one to be cloned (Bender* et al.* 2019, Vuckovic *et al.* 2019). However, mAChR M5 appears to have many important roles which makes it an attractive therapeutic target (Berizzi* et al.* 2016, Fujii* et al.* 2017, Vuckovic* et al.* 2019). mAChRs are present in the male reproductive system; in particular, M1–M4 mAChRs subtypes are found in efferent ductules, epididymides, vas deferens, seminal vesicles, and the prostate (Lucas* et al.* 2008). The mRNAs of M1–M5 mAChRs have been identified in rat testis Sertoli cells (Borges* et al.* 2001). mAChR antagonists are reported to disrupt male fertility (defects in efferent ductules, epididymides, vas deferens, and seminal vesicles) which indicates that mAChRs play vital roles in male reproduction (Lucas et al. 2008). Meanwhile, the roles of mAChRs in spermatogenesis and the underlying mechanisms are not understood.

Spermatogenesis is a complex process that involves meiosis and differentiation of germ cells from spermatogonia to spermatocytes, then on to spermatids (Zhou* et al.* 2016, Wang* et al.* 2018, Sohni* et al.* 2019). During spermatogenesis, the developing germ cells migrate progressively from the basal to the adluminal compartment with extensive junction restructuring between germ cell-Sertoli cell or Sertoli cell-Sertoli cell of the seminiferous epithelium. There are three major types of junctions found in the testis between germ cells and Sertoli cells, and among Sertoli cells: anchoring junctions (adherens junctions; AJs), tight junctions (TJs), and gap junctions (GJs) (Skinner 1991, Jegou 1993). It has been reported that Sertoli and germ cells develop an intimate and elaborate bidirectional trafficking system to communicate together through paracrine factors and signaling molecules that have been reported to play important roles in the regulation of cell–cell junction restructuring. Many investigations found that the following play vital roles in the cell–cell junctions: proteins like occludin, zonula occludens-1, claudin-11, and filamin A (Chung* et al.* 1999, Su* et al.* 2012); proteases and protease inhibitors such as cathepsin L, tryptase, cystatin C, and a2-macroglobulin (Mruk* et al.* 1997, Wong* et al.* 2000); cytokines such as transforming growth factor (TGF) b2 and TGFb3; kinases and phosphatases such as myotubularin (Li* et al.* 2001); small GTPases, such as Cdc42, N-Ras, Rac2, and RhoB (Lui* et al.* 2003*a,b*); transcriptional regulation (Lui *&* Cheng2012, Lie* et al.* 2013); signaling pathways such as the nitric oxide synthase (NOS)-cGMP-protein kinase G (PRKG)-b-catenin (CATNB) pathway (Lee* et al.* 2005), FAK pathway (Lie* et al.* 2013), and many other pathways (Lie* et al.* 2013, Li* et al.* 2018*a,b*, Wen* et al.* 2018*c*).

Although it is well known that during the development of spermatogonial cells into spermatids the destructuring and restructuring of cell–cell junctions takes place, the intriguing cross-talk mechanisms of regulation of this restructuring are not yet fully understood. In the current study, we report that the gene knockdown of M5 disrupted mouse spermatogenesis and damaged the actin-based cytoskeleton and many types of junction proteins in both Sertoli cells (TM4) and germ cells (C18-4); however, the knockdown of M1 or M3 had little effect on spermatogenesis even though there are many similarities between mAChRs M1, M3, and M5. Phldb2 (pleckstrin homology-like domain, family B, member 2, alternatively called LL5β) not only plays important roles in acetylcholine receptor (AChR) aggregation in the postsynaptic membrane, it is also involved in cell adhesion formation and extracellular matrix formation (Stehbens* et al.* 2014, Lim* et al.* 2016, Xie* et al.* 2019). Knockdown of M5 decreased PHLDB2 expression in both germ cells and Sertoli cells. Phldb2 may regulate cytoskeleton (actin) and other junctional proteins to control both BTB and ES formation to regulate spermatogenesis. The aim of this investigation was to explore the role of M5 in spermatogenesis, and where the PHLDB2 in the regulation of the BTB and ES in this process.

## Materials and methods

### Mice

All procedures involving live mice were performed in accordance with the NIH Guide for the Care and Use of Laboratory Animals and the protocols approved by the Institute of Animal Sciences, Chinese Academy of Agricultural Sciences Animal Care and Use Committee (2018AICAAS1002). ICR mice were used in this investigation. Testes from 1, 2, 3 and 6 weeks of age-old male ICR mice were collected for the detection of M1, M3, and M5 by immunofluorescence staining.

### Production of lentivirus

Lentivirus production was performed as described previously (Shen* et al.* 2019). Lenti-*shM5, shM1, shM3,* and *shNC* were cloned using the lentivirus-*shNC* vector as a backbone (Supplementary Fig. 1A, see section on supplementary materials given at the end of this article). There were three knockdown shRNAs at three different positions for each gene. The sequences for each gene (M1, M3, and M5) and NC are listed below:

shNC (5’–3’): TTCTCCGAACGTGTCACGT

shM1 #1 (5’–3’): GCATTCATCGGGATCACCACA

shM1 #2 (5’–3’): GGCCTACAGCTGGAAAGAAGA

shM1 #3 (5’–3’): GGACACCATATAACATCATGG

shM3 #1 (5’–3’): GCAACATCCTTGTCATTGTGG

shM 3 #2 (5’–3’): GCAGTGACAGTTGGAATAACA

shM 3 #3 (5’–3’): GGCCCAGAAGAGTATGGATGA

shM 5 #1 (5’–3’): GGAGTCTTATCACAATGAAAC

shM 5 #2 (5’–3’): GGACTCCTTATAACATCATGG

shM 5 #3 (5’–3’): GGACCCAGGAGACAAACAATG

The efficiency and specificity of shRNA knockdown were determined by transfecting into 293T cells using Lipofectamine 2000 (Invitrogen; #11668-027), followed by analysis at 60 h post-transfection by qPCR. Lentivirus production was then performed as shown in Supplementary Fig. 1B. Briefly, the lentiviral DNA was cotransfected with packaging plasmid pG-P1-VSVG, pG-P2-REV, and pG-P3-RRE HEK293T cells using RNAi-mate (GenePharma, Shanghai, China). The medium containing lentivirus was collected at 72 h post-transfection, pooled, filtered through a 0.2-μm filter, and concentrated using an ultracentrifuge at 85 000 ***g*** for 2 h (4°C). The virus was washed once and then resuspended in PBS. Approximately 10^9^ infectious viral particles/mL were obtained.

### *In vivo* virus grafting and sample collection

*In vivo* virus grafting was performed as previously described (Shen* et al.* 2019). In the current investigation, 5-week-old ICR male mice were used, because the pubertal period is a crucial window for testis development and spermatogenesis. Briefly, 5-week-old ICR male mice were anesthetized with isofluorane. Microinjections were performed using 26-gauge needles connected to a 100 μL syringe. Virus (3 μl with titer greater than 3×10^8^/mL) for each position and for each shRNA (in total 9 μL with a titer >9×10^8^/mL for shNC, or shM1, or shM3, or shM5 individually) were mixed and then injected into the testes. Ten days post-viral grafting, mice were deeply anesthetized with pentobarbital and the testes were collected. One testicle was fresh frozen in liquid nitrogen then total RNA was extracted for RNA-seq analysis or total protein was isolated for Western blotting (WB). The second testicle was fixed in 4% paraformaldehyde (PFA). Subsequently, the tissues were processed to be embedded in paraffin wax for immunochemical analyses as reported in our previous articles (Zhao* et al.* 2020*a*).

### Cell culture, transfection, and growth on cover slips

The TM4 cell line (mouse Sertoli cells; purchased from ATCC) was cultured in DMEM supplemented with 10% fetal bovine serum (FBS; Gibco; Thermo Fisher Scientific, Inc.) at 37°C in 5% CO_2_ (Hofmann *et al.* 2005). The C18-4 cell line (mouse spermatogonia stem cells; Donated by Dr Wenxian Zeng, Northwest A&F University) was held in DMEM/F12 (Gibco) supplemented with 10% (FBS), 2 mM L-glutamine (Invitrogen), and 100 U/mL penicillin and streptomycin (Invitrogen) (Hofmann* et al.* 2005, He* et al.* 2009, Li* et al.* 2018*c*). The cells were transfected with shRNA in 6-well plates. Similarly, three respective shRNAs for each gene were mixed together (titer >3×10^8^/mL) with RNAi-mate for the transfection for both C18-4 and TM4 cells. The transfection medium was changed after 12 h. Stable transfected cells were cultured in a similar manner to the non-transfected cells in their respective media. The transfected cells were plated on the coverslips in a 6-well plate for 2 days, after which the coverslips with the cells were collected and fixed in 4% PFA for immunofluorescence staining.

### RNA isolation and RNA-seq analyses as reported in our earlier article (Zhang *et al.* 2016)

Briefly, total RNA was isolated using TRIzol Reagent (Invitrogen) and purified using a Pure-Link1 RNA Mini Kit (Cat: 12183018A; Life Technologies) following the manufacturer's protocol. Total RNA samples were first treated with DNase I to degrade any possible DNA contamination. Then, the mRNA was enriched using oligo(dT) magnetic beads. Mixed with the fragmentation buffer, the mRNA was broken into short fragments (about 200 bp), after which, the first strand of cDNA was synthesized using a random hexamer-primer. Buffer, dNTPs, RNase H, and DNA polymerase I were added to synthesize the second strand. The double-strand cDNA was purified with magnetic beads. Subsequently, 3'-end single nucleotide A (adenine) addition was performed. Finally, sequencing adaptors were ligated to the fragments. The fragments were enriched by PCR amplification. During the QC step, an Agilent 2100 Bioanaylzer and ABI StepOnePlus Real-Time PCR System were used to qualify and quantify the sample library. The library products were prepared for sequencing in an Illumina HiSeqTM 2500. The reads were mapped to reference genes using SOAPaligner (v. 2.20) with a maximum of two nucleotide mismatches allowed at the parameters of '-m 0 -× 1000 -s 40 -l 35 -v 3 -r 2'. The read number of each gene was transformed into RPKM (reads per kilo bases per million reads), and then differentially expressed genes were identified using the DEGseq package and the MARS (MA-plot-based method with random sampling model) method. The threshold was set as FDR ≤0.001 and an absolute value of log_2_ ratio ≥1 to judge the significance of the difference in gene expression. To identify the main sources of variation in the dataset (PCA), we employed the FPKM values as the input for principal component analysis using the FactorMiner R package. The significance of the principal components was obtained with the Seurat package via a permutation test, after 1000 randomized samplings. Then, the data were analyzed by GO enrichment, KEGG enrichment or Metascape (http://metascape.org/gp/index.html#/main/step1).

### Western blotting

Western blotting analysis of proteins was carried out as previously reported (Zhang* et al.* 2016, Zhao* et al.* 2020*b*). Briefly, testicular tissue samples were lysed in RIPA buffer containing the protease inhibitor cocktail from Sangong Biotech, Ltd. (Shanghai, China). Protein concentration was determined using a BCA kit (Beyotime Institute of Biotechnology, Shanghai, China). Goat anti-glyceraldehyde 3-phosphate dehydrogenase (GAPDH; Cat #: sc-48166, Santa Cruz Biotechnology, Inc., Dallas, Texas, USA) was used as a loading control. The remaining primary antibodies (Abs) were purchased from Abcam or Beijing Biosynthesis Biotechnology CO., LTD, (Beijing, China; Supplementary Table 1). Secondary donkey anti-goat Ab (Cat no.: A0181) was purchased from Beyotime Institute of Biotechnology, and goat anti-rabbit (Cat no.: A24531) Abs were bought from Novex^®^ by Life Technologies. Fifty micrograms of total protein per sample were loaded onto 10% SDS polyacrylamide electrophoresis gels. The gels were transferred to a polyvinylidene fluoride (PVDF) membrane at 300 mA for 2.5 h at 4°C. The membranes were then blocked with 5% BSA for 1 h at RT, followed by three washes with 0.1% Tween-20 in TBS (TBST). The membranes were incubated with primary Abs diluted with 1:500 in TBST with 1% BSA overnight at 4°C. After three washes with TBST, the blots were incubated with the HRP-labeled secondary goat anti-rabbit or donkey anti-goat Ab respectively for 1 h at RT. After three washes, the blots were imaged. The bands were quantified using Image-J software. The intensity of the specific protein band was normalized to actin first, then, the data were normalized to the control. The experiment was repeated > six times.

### Detection of protein levels and location in testis using immunofluorescence staining

The methodology for immunofluorescence staining of testicular samples is reported in our recent publications (Zhang* et al.* 2016, Zhao* et al.* 2020*b*). Sections of testicular tissue (5 µm) were prepared and subjected to antigen retrieval and immunostaining as previously described (Wang* et al.* 2018). Briefly, sections were first blocked with normal goat serum in PBS, followed by incubation with primary Abs (Supplementary Table 1; 1:100 in PBS-0.5% Triton X-100; Bioss Co. Ltd. Beijing, PR China) at 4°C overnight. After a brief wash, sections were incubated with an Alexa 546-labeled goat anti-rabbit secondary Ab (1:100 in PBS; Molecular Probes) at RT for 30 min and then counterstained with 4',DAPI. The stained sections were examined using a Leica Laser Scanning Confocal Microscope (LEICA TCS SP5 II, Germany). Ten animal samples from each treatment group were analyzed. Positively stained cells were counted. A minimum of 1000 cells were counted for each sample of each experiment. The data were then normalized to the control.

### Immunofluorescence staining for cells on cover slips

The cells on coverslips were fixed with 4% PFA overnight (Zhao* et al.* 2020b). Subsequently, the cells were treated with 2% Triton X-100 in PBS for 30 min. After three washes with PBS, the cells were blocked with normal goat serum in PBS, followed by incubation with primary Abs (Supplementary Table 1) at 4°C overnight. After a brief wash, sections were incubated with an Alexa 546-labeled goat anti-rabbit secondary Ab (1:100 in PBS) at RT for 30 min and then counterstained with DAPI. For actin formation, the cells were incubated with Phalloidin-TRITC for 1 h at RT, then counterstained with DAPI. The stained sections were examined using a Leica Laser Scanning Confocal Microscope (LEICA TCS SP5 II, Germany). The experiments were repeated at least six times.

### Statistical analysis

Data were statistically analyzed using the ANOVA function in SPSS statistical software (IBM Co.). Comparisons between groups were tested by one-Way ANOVA analysis and the LSD test. All groups were compared with each other for every parameter (mean ± s.e.m.). Differences were considered significant at *P*  < 0.05.

## Results

### M5 knockdown disrupted mouse spermatogenesis, while M1 and M3 knockdown did not

The expression of M5, M1, and M3 mACHRs has been detected in mouse testes using specific antibodies (Supplementary Fig. 2). M5 and M1 receptor expression levels were increased in murine testes between 1 and 6 weeks of age. However, M3 receptor expression was low in 1-week-old mouse testes while it was elevated at a consistent level between 3 and 6 weeks of age. These three receptors are expressed in Sertoli cells and germ cells. M5 and M1 receptors are mainly expressed in the perinuclei region, while M3 is mainly expressed in the nuclei, which is constant with the findings of Lucas et al (2008). Moreover, M1 is mainly present in spermatids of 6-week-old mice, while M5 and M3 are present mainly in the early stages of germ cells (spermatogonia or spermatocytes; Supplementary Fig. 2).

**Figure 1:**
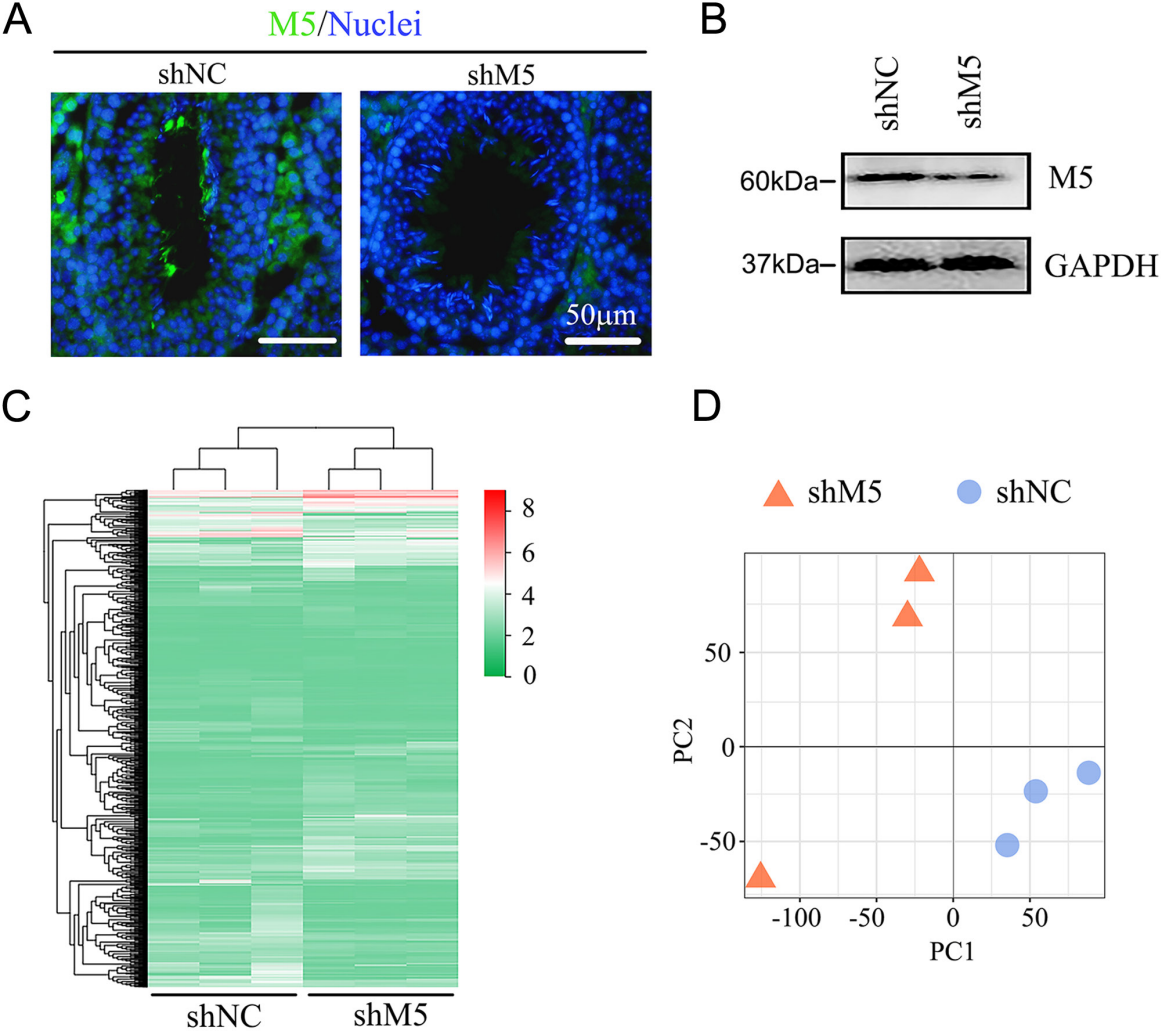
M5 knockdown by shRNA in mouse testes disrupted spermatogenesis *in vivo*. (A) The protein level of M5 was detected by immunofluorescence staining (IHF) in mouse testes after treatment with M5 shRNA for 10 days. Scale bar: 50 µm. (B) The protein level of M5 was detected by Western blotting (WB) in mouse testes after treatment with shRNA for 10 days. (C) Gene expression heatmap of mouse testicular samples after shRNA treatment for 10 days. (D) Principal component analysis (PCA) for gene expression in mouse testes.

**Figure 2:**
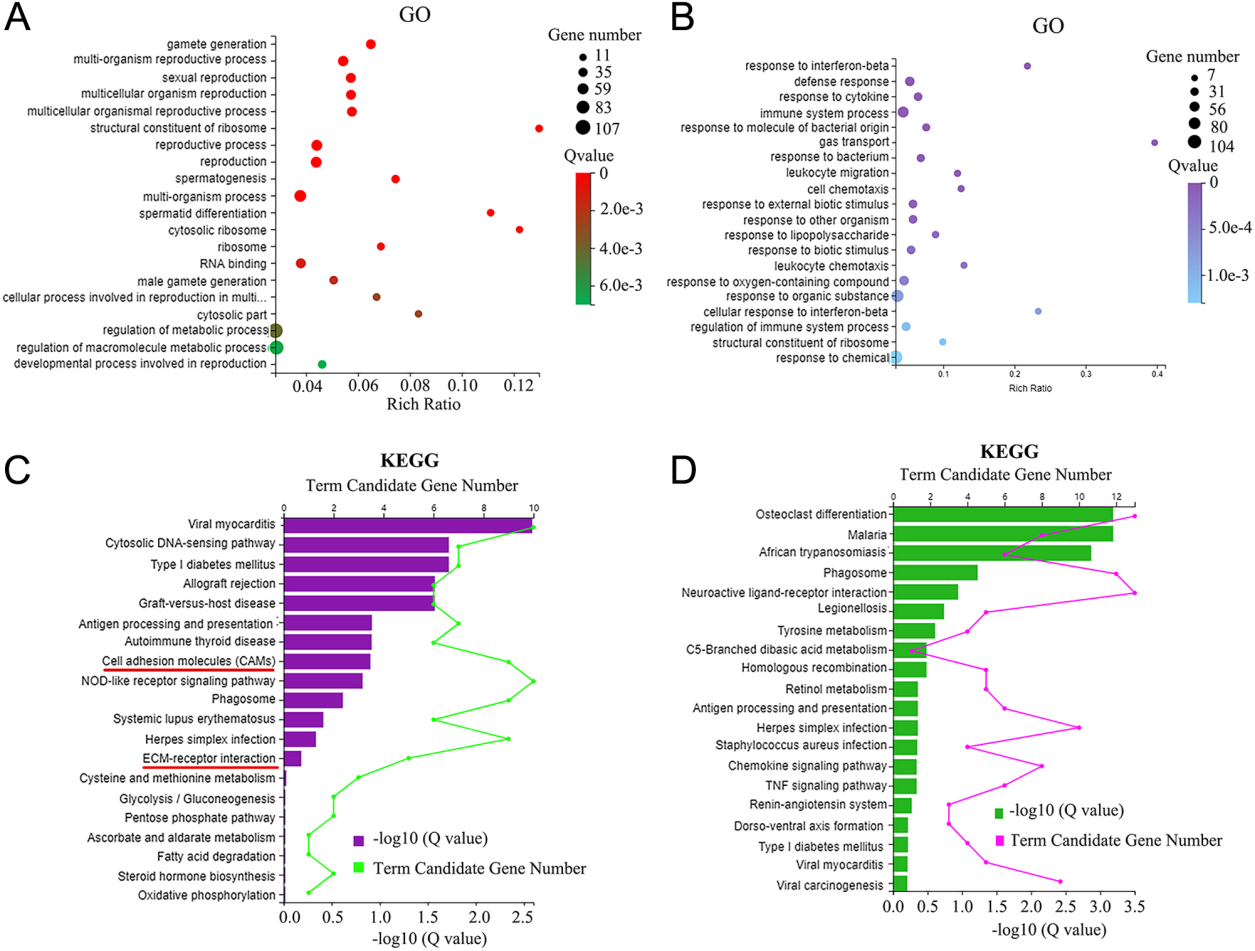
Enrichment analysis for RNA-seq data of mouse testes. (A) Gene ontology (GO) enrichment analysis of the genes decreased by shM5 treatment in mouse testes. (B) GO enrichment analysis of the genes increased by shM5 treatment in mouse testes. (C) Kyoto Encyclopedia of Genes and Genomes (KEGG) enrichment analysis of the genes decreased by shM5 treatment in mouse testes. (D) KEGG enrichment analysis of the genes increased by shM5 treatment in mouse testes.

In order to differentiate the function of M5 from M1 or M3, as they all couple to Gq_/11_, the expression of these receptors was modified in mouse testes during the current investigation. After M5 knockdown using shRNA, the protein level of the M5 receptor was decreased (Fig. 1A and B; Supplementary Fig. 3). There was also some staining for M5 on the Leydig cells which indicated that these cells also expressed M5. The gene expression profile of mouse testes was then determined by RNA-seq analysis. Compared to the control (shNC), 661 genes were increased while 622 genes were decreased in shM5 mouse testis samples (Fig. 1C). Principal component analysis (PCA) showed that the shM5 and shNC groups were clearly separated (Fig. 1D). The genes decreased by shM5 were mainly enriched in the gamete generation, spermatogenesis, and male reproduction functional pathways in gene ontology (GO) enrichment analysis (Fig. 2A), while those genes increased by shM5 were enriched in others not related to spermatogenesis signaling pathways (Fig. 2B). In order to search more deeply for the mechanisms of shM5 knockdown disruption of spermatogenesis, Kyoto Encyclopedia of Genes and Genomes (KEGG) enrichment analysis was performed. There were two signaling pathways 'cell adhesion molecules (CAMs)' and 'ECM-receptor interaction' that were significantly enriched in the decreased genes (Fig. 2C), while they were not found in the increased genes (Fig. 2D). As we know, cell–cell junctions (especially in the BTB) play vital roles in spermatogenesis and male fertility (Lee* et al.* 2005, Lui *&* Cheng 2012, Li* et al.* 2018*b*, Wen* et al.* 2018*c*). The data suggested that shM5 may damage cell–cell junctions in the testis to disrupt spermatogenesis.

However, shM1 had some effect and shM3 had little effect on spermatogenesis (Supplementary Figs 4 and 5). There were 263 genes increased and 550 genes decreased by shM1 (Supplementary Fig. 4A). Furthermore, shM1 and shNC were clearly separated by PCA analysis (Supplementary Fig. 4B). A total of 404 genes were increased and 724 genes were decreased by shM3 and PCA analysis showed that shM3 and shNC were also clearly separated (Supplementary Fig. 5A and B). GO enrichment analysis showed that there was one signaling pathway 'gamete generation' enriched in shM1 decreased genes (Supplementary Fig. 4C). In addition, no pathways related to spermatogenesis or male fertility were enriched by shM3 knockdown genes (Supplementary Fig. 5C). The data here suggested that M1 or M3 may have a different role from M5 in mouse spermatogenesis.

**Figure 3:**
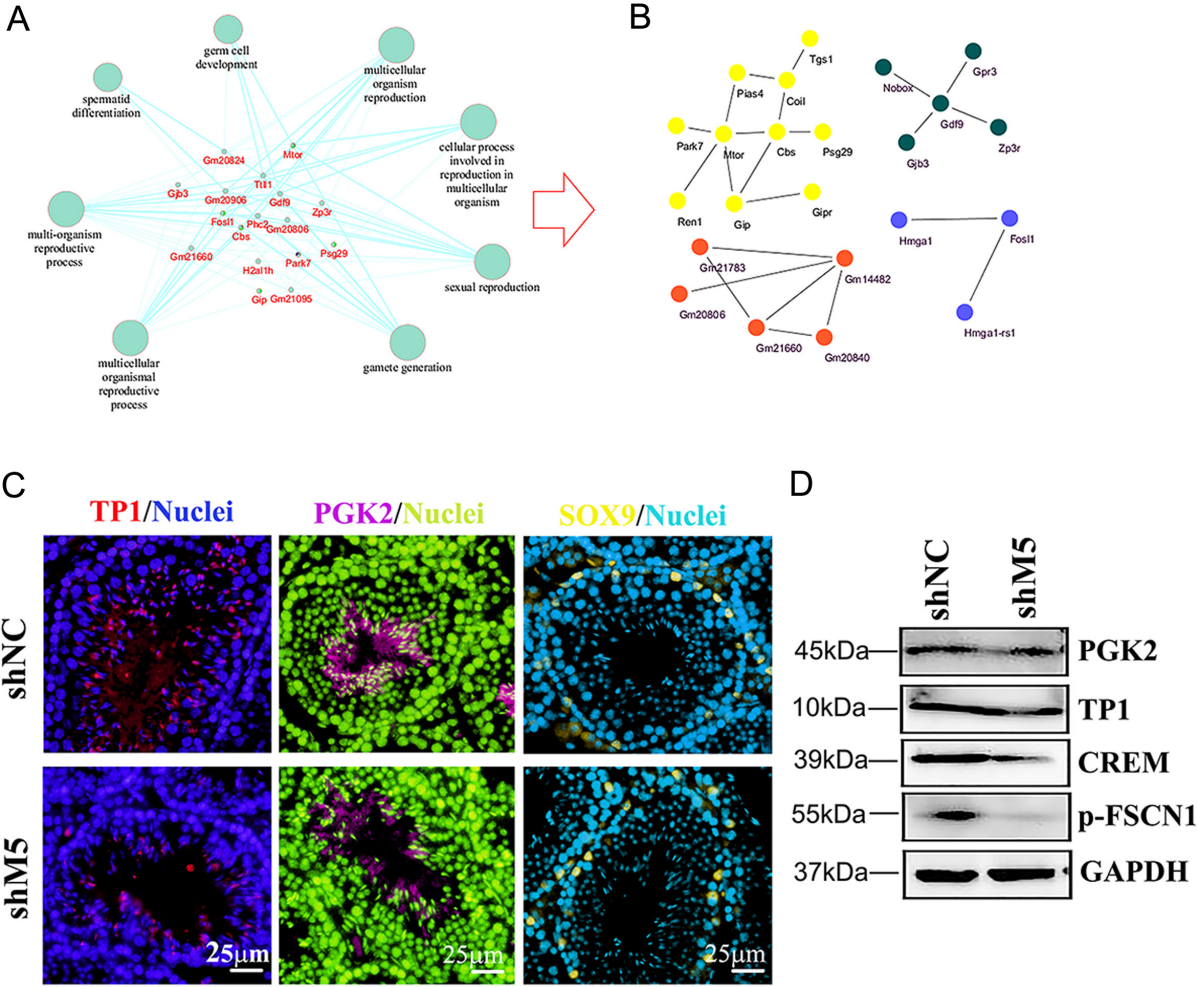
Network analysis of the genes related to spermatogenesis and male fertility in mouse testes. (A) The network of genes related to spermatogenesis and male fertility, and the signaling pathways enriched by GO analysis. (B) The network of genes related to spermatogenesis and male fertility enriched by GO analysis. (C) The protein level of TP1, PGK2, and SOX9 was detected by IHF in mouse testes after shM5 treatment. Scale bar: 25 µm. (D) The protein level of TP1, PGK2, CREM, and p-FSCN1 was detected by WB in mouse testes after shM5 treatment.

Using GO enrichment analysis, the signaling pathways related to spermatogenesis and male reproduction for the shM5 group were separated and analyzed for their protein–protein network. The proteins in these pathways were connected together with the above-mentioned signaling pathways (Fig. 3A). These proteins could be divided into three groups as shown in Fig. 3B. Furthermore, we found that the levels of proteins important to spermatogenesis (Lee* et al.* 2005, Li* et al.* 2018*a*, Wen* et al.* 2018*b*) (TP1, PGK2, CREM, and p-FSCN1) were decreased in shM5 mouse testis samples (Fig. 3C and D, Supplementary Fig. 6). Meanwhile, Sertoli cell marker SOX9 remained unchanged by shM5 (Fig. 3C). The data further suggested that shM5 upset spermatogenesis.

**Figure 4:**
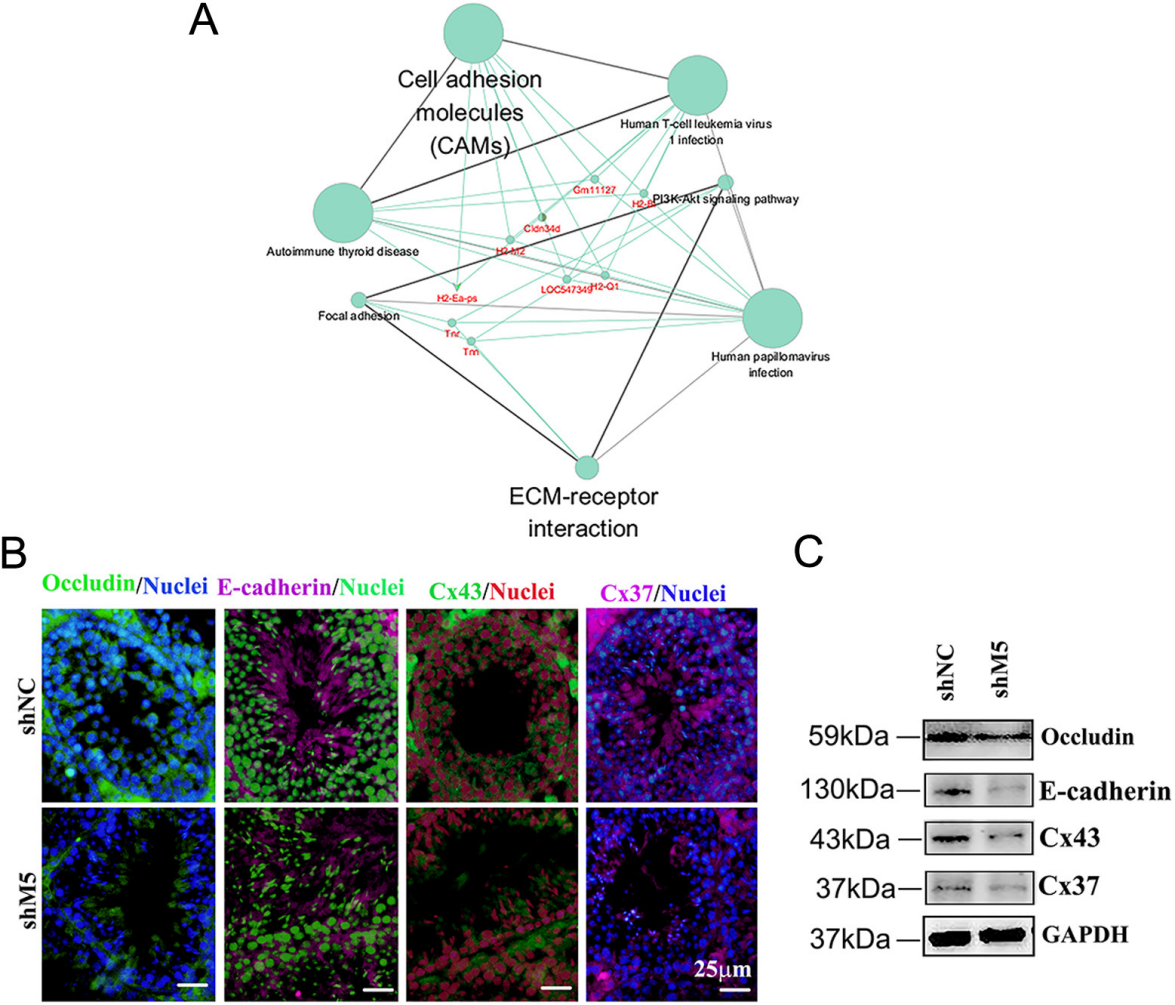
Network analysis of the genes related to cell–cell junctions in mouse testes. (A) The network of genes related to cell–cell junctions, and the signaling pathways enriched by GO analysis. (B) Protein levels of occludin, claudin, Cx43, and Cx37 were detected by IHF in mouse testes after shM5 treatment. Scale bar: 25 µm. (C) Protein level of occludin detected by WB in mouse testes after shM5 treatment.

The genes (decreased by shM5) in 'cell adhesion molecules' and 'ECM-receptor interaction' pathways from KEGG enrichment analysis were further analyzed to determine their interactive network. These genes were connected together with the signaling pathways (Fig. 4A). Furthermore, the protein levels of many cell–cell junction proteins such as claudin, occludin, Cx43, Cx37, JAM1, ZO-1, E-cadherin, and catenin were determined in mouse testis samples. Results showed that the cellular localization of occludin, E-cadherin, Cx37, and Cx43 were changed by shM5 (Fig. 4B and C; Supplementary Fig. 6). The data here further indicated that shM5 may damage cell–cell junctions to disrupt spermatogenesis.

### Knockdown M5 decreased cell–cell junction proteins in spermatogonia stem cells

**Figure 5:**
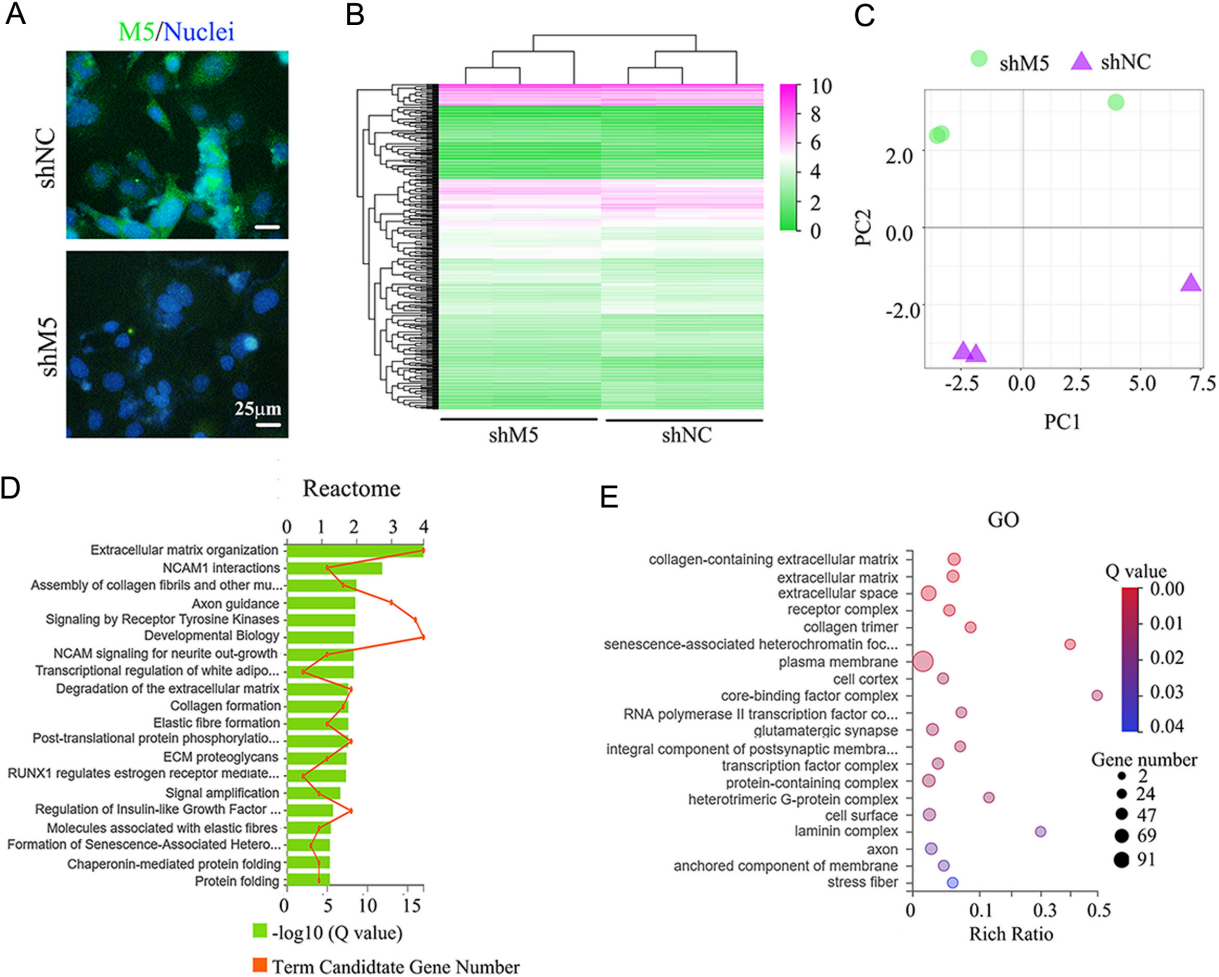
RNA-seq analysis of mouse spermatogonia cells (C18-4) after shM5 treatment. (A) Protein level of M5 detected by IHF in C18-4 cells after shM5 treatment. Scale bar: 25 µm. (B) Gene expression heatmap of C18-4 cells after shM5 treatment. (C) PCA analysis for gene expression of C18-4 cells. (D) Reactome enrichment analysis of the genes decreased by shM5 treatment in C18-4 cells. (E) GO enrichment analysis of the genes decreased by shM5 treatment in C18-4 cells.

**Figure 6:**
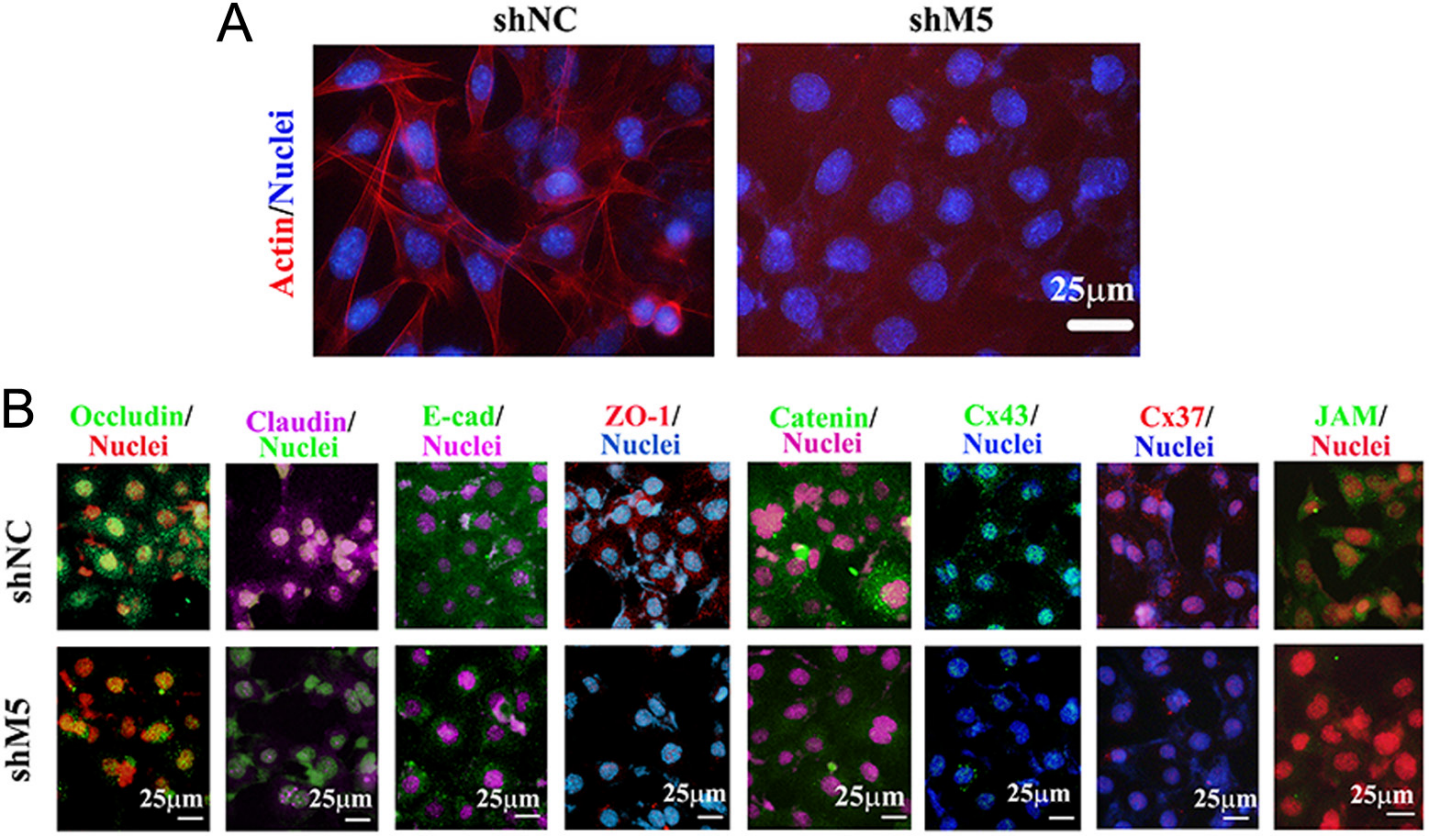
shM5 disrupted the blood–testis barrier and ectoplasmic specialization (ES) proteins in C18-4. (A) Status of the cell cytoskeleton (actin) in C18-4 cells was detected by IHF after shM5 treatment. (B) Protein levels of the blood-testis barrier (BTB) and ectoplasmic specializations (ES) specific proteins occludin, claudin, E-cadherin, ZO-1, catenin, Cx43, Cx37, and JAM1 were detected by IHF in C18-4 cells after shM5 treatment. Scale bar: 25 µm.

To further search for the M5 mechanism during spermatogenesis, M5 expression was modified in spermatogonia stem cells (C18-4 cell line) (Hofmann* et al.* 2005, He* et al.* 2009) and Sertoli cells (TM4 cell line) (Ge* et al.* 2018). The protein level of M5 in C18-4 cells was decreased by shM5 (Fig. 5A) and the gene expression profile in C18-4 cells after shM5 treatment was significantly altered. An increase was seen in 239 genes, while 261 genes were decreased by shM5 compared to shNC (Fig. 5B). The PCA data showed that shM5 was clearly separated from shNC (Fig. 5C). Reactome and GO enrichment analyses showed that many signaling pathways were related to the extracellular matrix and cell adhesion for the genes decreased by shM5 (Fig. 5D and E), but not for those genes that were increased (Supplementary Fig. 7A and B). Furthermore, cell cytoskeleton (actin) was detected in C18-4 cells after shM5 treatment, and it was noteworthy that actin formation was dramatically damaged by shM5 (Fig. 6A). Subsequently, the cell–cell junction proteins were determined to confirm the disruption to the cytoskeleton. All eight cell–cell junction proteins occludin, claudin, JAM1, Cx37, Cx43, E-cadherin, ZO-1, and catenin were decreased by shM5 compared to shNC (Fig. 6B; Supplementary Fig. 8). The data here suggested that M5 plays a vital role in cell–cell junction formation in germ cells.

### M5 knockdown decreased cell–cell junction proteins in Sertoli cells

**Figure 7:**
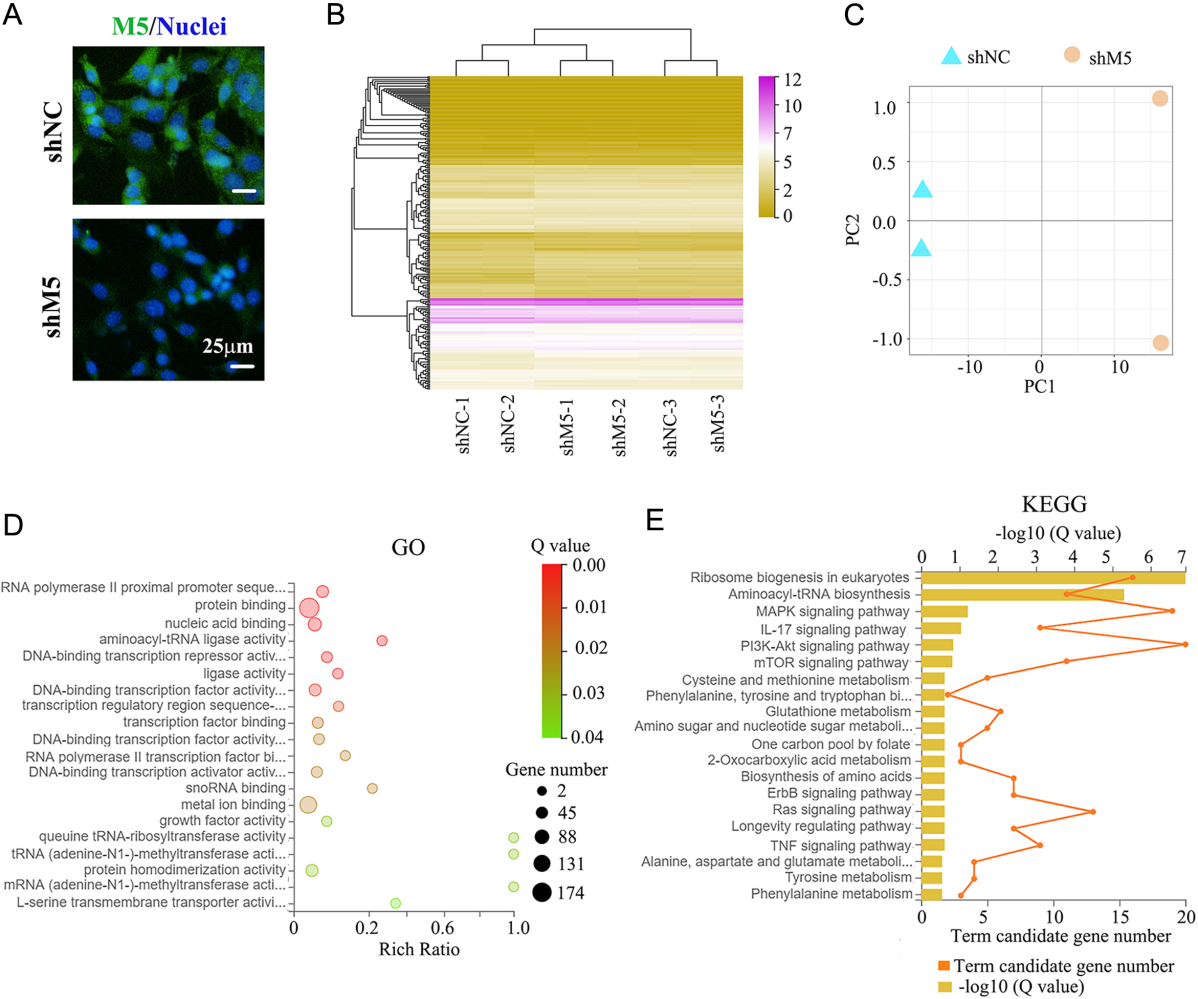
RNA-seq analysis of mouse Sertoli cells (TM4) after shM5 treatment. (A) Protein level of M5 detected by IHF in TM4 cells after shRNA treatment. Scale bar: 25 µm. (B) Gene expression heatmap of TM4 cells after shRNA treatment. (C) PCA analysis for the gene expression of TM4 cells. (D) Reactome enrichment analysis of the genes decreased by shM5 treatment in TM4 cells. (E) GO enrichment analysis of the genes decreased by shM5 treatment in TM4 cells.

**Figure 8:**
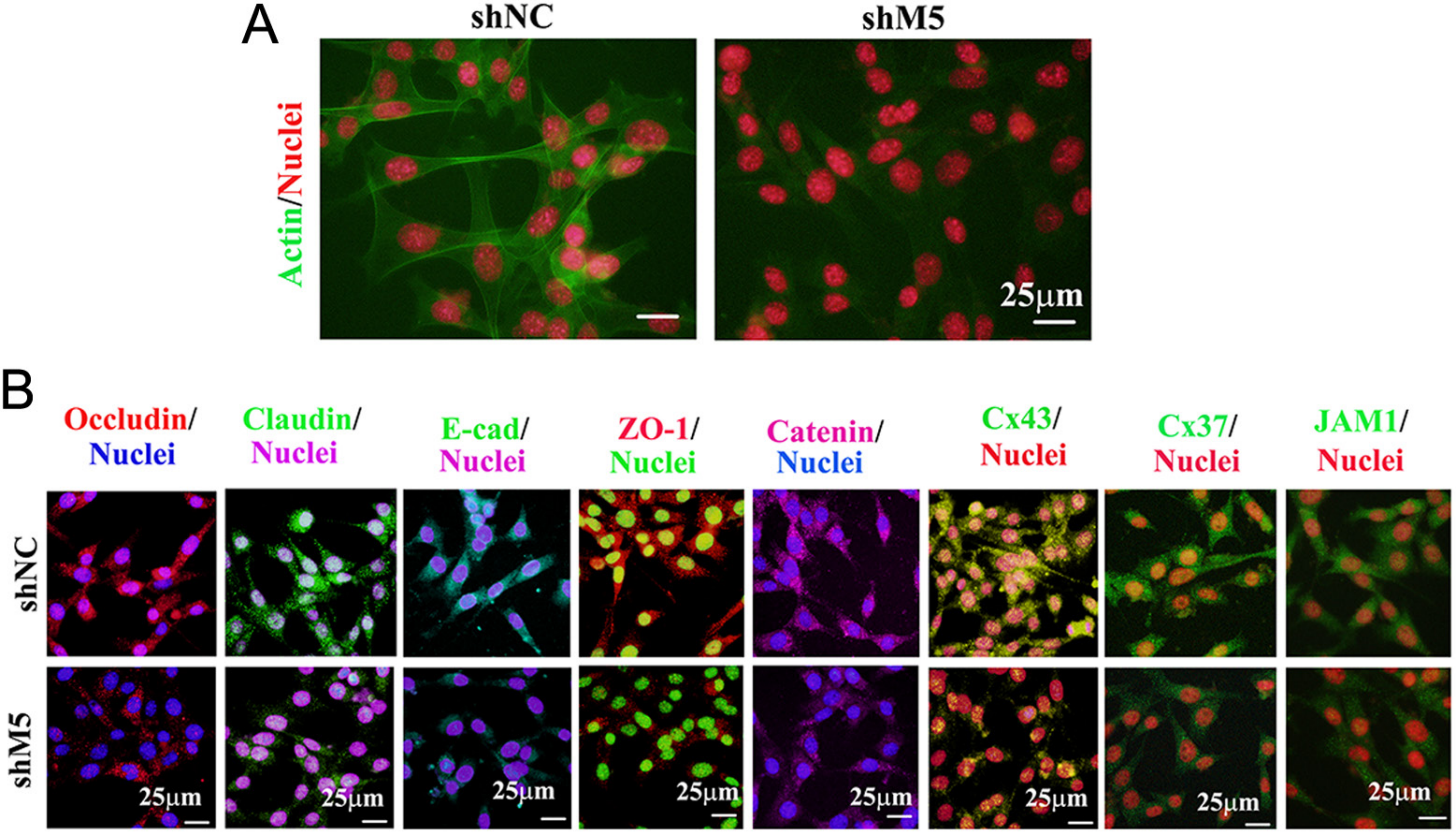
shM5 disrupted the blood–testis barrier and ectoplasmic specialization (ES) proteins in C18-4. (A) Status of the cell cytoskeleton (actin) in TM4 cells was detected by IHF after shM5 treatment. (B) Protein levels of the BTB and ES specific proteins occludin, claudin, E-cadherin, ZO-1, catenin, Cx43, Cx37, and JAM1 were detected by IHF in TM4 cells after shM5 treatment. Scale bar: 25 µm.

M5 protein levels in Sertoli cells (TM4 cell line) were decreased by shM5 (Fig. 7A). shM5 significantly changed the gene expression profile in TM4 cells. In total, 90 genes were increased and 566 genes were decreased by shM5 in TM4 cells (Fig. 7B). PCA analysis found that shNC and shM5 could be clearly separated (Fig. 7C). GO enrichment analysis found that 174 out of 566 genes (decreased genes) were enriched into 'protein binding' functional signaling pathways which suggested that Sertoli cell junctions may be affected by shM5 (Fig. 7D), but not for those genes that were increased (Supplementary Fig. 6C and D). The next step was to determine the cytoskeleton (actin formation) in TM4 cells. It was found that actin formation was disrupted by shM5 in TM4 cells (Fig. 8A). At the same time, the protein levels of the eight cell junctional proteins occludin, claudin, JAM1, Cx37, Cx43, E-cadherin, ZO-1, and catenin were significantly reduced by shM5 in Sertoli cells (Fig. 8B; Supplementary Fig. 8). Furthermore, there were a few functional pathways related to Sertoli cell functions such as the MAPK signaling pathway, PI3K-AKT pathway, and mTOR pathway that have been enriched in KEGG analysis for the genes decreased by shM5 in TM4 cells (Fig. 7E) (Ni* et al.* 2019). The data here suggested that shM5 upset the function of Sertoli cells, in particular, the cell junctions.

### Deep analysis discovered the overlap of functional pathways in both C18-4 and TM4 cells caused by shM5

**Figure 9:**
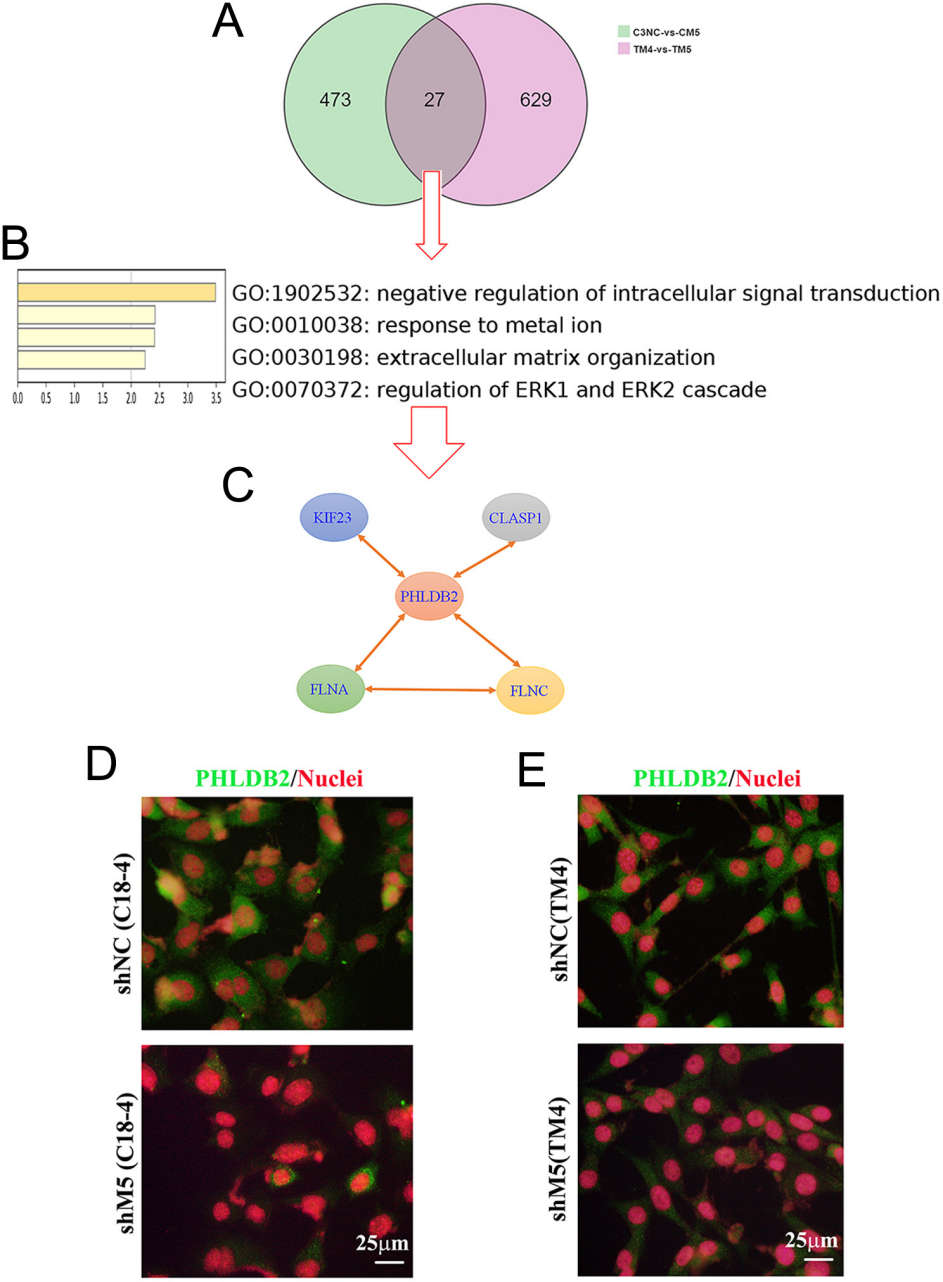
Phldb2 pathway involved in the disruption of the blood-testis barrier (BTB) and ectoplasmic specialization by knockdown M5 in C18-4 and TM4 cells *in vitro*. (A) Summary of gene expression after shM5 treatment in C18-4 and TM4 cells. (B) The enrichment data of the 27 genes (overlay in both C18-4 and TM4 cells) by Metascape online. (C) Gene network of the PHLDB2 signaling pathway. (D) Protein levels of PHLDB2 in C18-4 cells after shM5 treatment. Scale bar: 25 µm. (E) Protein levels of PHLDB2 in TM4 cells after shM5 treatment. Scale bar: 25 µm.

Twenty-seven genes overlapped between C18-4 and TM4 cells by shM5 (Fig. 9A). Enrichment analysis of those 27 genes showed that they play important roles in intracellular signaling transduction and the extracellular matrix (Fig. 9B). One of the genes, Phldb2, not only plays an important role in AChR aggregation in the postsynaptic membrane (Xie* et al.* 2019) but it is also involved in cell adhesion formation and extracellular matrix formation (Stehbens* et al.* 2014, Lim* et al.* 2016). Phldb2 interacts with CLASP and filamins during its involvement in focal adhesion and extracellular matrix turnover (Fig. 9C) (Su* et al.* 2012, Stehbens* et al.* 2014). The expression of Phldb2 was decreased in both C18-4 and TM4 cells. Furthermore, the protein levels of PHLDB2 were significantly decreased by shM5 in C18-4 cells (Fig. 9D; Supplementary Fig. 8) and TM4 cells (Fig. 9E; Supplementary Fig. 8).

## Discussion

mAChRs include five subtypes (M1–M5) which are known to play various roles in the physiology and pathophysiology of different autocrine and neuronal systems. Activation of mAChRs is known to be involved in cell proliferation, differentiation, growth, and other functions in male reproductive systems. This activation could release EGF receptor ligands to bind to EGFR and could activate the extracellular signal-regulated kinase (ERK)1/2 pathway (Lucas* et al.* 2008) to stimulate Sertoli cell proliferation. However, there are currently no reports regarding the activation of mAChR on spermatogenesis. Although there are many similarities between M1, M3, and M5, their functions in mouse testes are very different. In the current investigation, we found that knockdown of M1 had some effect and knockdown of M3 had little effect on spermatogenesis, whereas M5 knockdown disrupted spermatogenesis and damaged the expression of proteins (claudin, occludin, Zonula occludens-1 (ZO-1), junctional adhesion molecules (JAM1), connexin 43 (Cx43), Cx37, E-cadherin, and catenin) in the blood–testis barrier (BTB) and ectoplasmic specializations (ES)).

Spermatogenesis is a choreographed process of diploid spermatogonia undergoing differentiation to produce haploid germ cells. During spermatogenesis, extensive remodeling at Sertoli cell–cell and Sertoli cell-germ cell interfaces takes place to accommodate the transport of developing germ cells across the BTB and adluminal compartment (Wen* et al.* 2018*b*). The BTB is made up of actin-based tight junctions (TJs) and gap junctions (GJs). The extracellular matrix (ECM) is involved in spermatogenesis by regulating the BTB since Sertoli cells are in physical contact with the basement membrane. Although the BTB plays vital role in the control of Sertoli cell–cell interactions, ES is a unique actin-rich AJ in the testes that regulates Sertoli cell-germ cell interactions. ES includes basal ES at the Sertoli cell–cell interface of the BTB, and the apical ES at the Sertoli–spermatid interface (Li* et al.* 2015). Actin-based cytoskeletons in Sertoli and germ cells play crucial roles in the regulation of homeostasis of the BTB and the cytoskeletal elements at the basal ES (Wen* et al.* 2018*a*). Even though there have been many recent reports that have highlighted the factors involved in the regulation of the BTB and ES (Li* et al.* 2015, Wen* et al.* 2018*a*), the intriguing cross-talk mechanism(s) between basal and apical ES are still not fully understood, because the restructuring of the BTB close to the basement membrane and the disruption of the apical tubulobulbar complex (TBC) at the luminal edge of the epithelium happen almost concurrently (Li* et al.* 2015). In the current investigation, we aimed to explore the underlying mechanisms regulating the basal and apical ES. We found that shM5 disrupted mouse spermatogenesis *in vivo* and damaged the actin-based cytoskeleton and many types of junction proteins in both Sertoli cells (TM4) and germ cells (C18-4). Claudin and occludin are major players in Sertoli cell TJs (McCabe* et al.* 2016). Cadherins, AJ transmembrane proteins, are reported to be present at the basal ES (Lee* et al.* 2003). Catenins and ZO-1, the peripheral adaptors of basal ES and TJs, are involved in the engagement/disengagement mechanism between basal ES and TJsto make the BTB one of the 'tightest' barriers in the mammalian body (Yan & Cheng 2005). Connexin 43, connexin 37, and JAMs have also been found to play important roles in cell–cell junctions (Zhang & Lui 2015, Li* et al.* 2016). In the current investigation, claudin, occludin, ZO-1, JAM1 Cx43, Cx37, E-cadherin, and catenin were found to be decreased by shM5 in spermatogonial cells (C18-4) and Sertoli cells (TM4). Our data suggested that M5 may be involved in ES and BTB restructuring to regulate spermatogenesis.

Phldb2 (pleckstrin homology-like domain, family B, member 2) is a PH domain-containing protein that is highly sensitive to phosphatidylinositol 3,4,5-triphosphate (PIP3) as well as PIP2 (Xie* et al.* 2019). Phldb2 plays important role in AChR aggregation in the postsynaptic membrane (Xie* et al.* 2019). Moreover, Phldb2 can associate with CLIP-associating proteins (CLASPs), Prickle 1, and Liprin α1 to be involved in focal adhesion disassembly and cell polarization and migration (Astro* et al.* 2014, Lansbergen* et al.* 2006, Lim* et al.* 2016). Podosomes are actin-rich, dynamic structures capable of remodeling the extracellular matrix (ECM) and have been found in many types of cells such as osteoclasts, macrophages, and epithelial cells. Podosomes present around AChR aggregates are called 'synaptic podosome' (Proszynski & Sanes 2013). The cytoskeleton- and membrane-associated protein Phldb2 is known to be one of the key components of synaptic podosomes, and its upset perturbs AChR clustering in cultured myotubes (Kishi* et al.* 2005). In the current study, we found that shM5 decreased Phldb2 expression in both germ cells and Sertoli cells. Therefore, Phldb2 may regulate the cytoskeleton (actin) and other junctional proteins (claudin, occludin, Cx43, ZO-1, cadherin, and catenin) to control BTB and ES formation that in turn affect spermatogenesis. Since Phldb2 interacts with AChR, the mAChR M5 knockdown may upset Phldb2 and disrupt the BTB and ES to damage spermatogenesis.

In summary, our investigation has elucidated a novel role for mAChR M5 in the regulation of spermatogenesis through Phldb2 regulation of the BTB and ES. Further studies on the cross-talk between M5 and Phldb2, and among Phldb2, BTB, and ES will shed light on our understanding of the mechanisms of M5 in the regulation of the BTB/ES and spermatogenesis.

## Supplementary Material

Supplemental Fig. 1 Structure of the lentivirus vector used in this study and its production protocol for subsequent infection of cells and animals.

Supplemental Fig. 2 Protein expression of muscarinic acetylcholine receptors (mAChRs) M1, M3, and M5 in mouse testes at the age of 1, 2, 3, and 6 weeks. Scale bar: 25 μm.

Supplemental Fig. 3 Quantitative data for IHF and WB of mouse testis samples. A Quantitative data for IHF of mouse testis samples (for M5 detection). B Quantitative data for WB of mouse testis samples (for M5 detection). (n=6/group). Data are presented as mean ± SEM. a, b indicate a significant difference among different treatments (p < 0.05).

Supplemental Fig. 4 Gene expression in mouse testes after M1 knockdown by short hairpin RNA (shRNA) in vivo. A Gene expression heatmap of mouse testis samples after M1 shRNA treatment for 10 days. B PCA analysis for the gene expression of mouse testes. C GO enrichment analysis of the genes decreased by shM1 treatment in mouse testes. D GO enrichment analysis of the genes increased by shM1 treatment in mouse testes. E KEGG enrichment analysis of the genes decreased by shM1 treatment in mouse testes. F KEGG enrichment analysis of the genes increased by shM1 treatment in mouse testes.

Supplemental Fig. 5 Gene expression in mouse testes after M3 knockdown by short hairpin RNA (shRNA) in vivo. A Gene expression heatmap of mouse testis samples after M3 shRNA treatment for 10 days. B PCA analysis for the gene expression of mouse testes. C GO enrichment analysis of the genes decreased by shM3 treatment in mouse testes. D GO enrichment analysis of the genes increased by shM3 treatment in mouse testes. E KEGG enrichment analysis of the genes decreased by shM3 treatment in mouse testes. F KEGG enrichment analysis of the genes increased by shM3 treatment in mouse testes.

Supplemental Fig. 6 Quantitative data for WB of mouse testis samples. A Quantitative data for WB of mouse testis samples for the detection of PGK2. B Quantitative data for WB of mouse testis samples for the detection of TP1. C Quantitative data for WB of mouse testis samples for the detection of CREM. D Quantitative data for WB of mouse testis samples for the detection of p-FSCN1. E Quantitative data for WB of mouse testis samples for the detection of occludin. (n=6/group). Data are presented as mean ± SEM. a, b indicate a significant difference among different treatments (p < 0.05).

Supplemental Fig. 7 The data summary of increased gene in C18-4 and TM4 cells after shM5 treatment in vitro. A Reactome enrichment analysis of the genes increased by shM5 treatment in C18-4 cells. B GO enrichment analysis of the genes increased by shM5 treatment in C18-4 cells. C Reactome enrichment analysis of the genes increased by shM5 treatment in TM4 cells. D GO enrichment analysis of the genes increased by shM5 treatment in TM4 cells.

Supplemental Fig. 8 Quantitative data for IHF of C18-4 and TM4 cell samples. A Quantitative data for IHF of C18-4 and TM4 cell samples for the detection of occludin. B Quantitative data for IHF of C18-4 and TM4 cell samples for the detection of claudin. C Quantitative data for IHF of C18-4 and TM4 cell samples for the detection of E-cad (E-cadherin). D Quantitative data for IHF of C18-4 and TM4 cell samples for the detection of ZO-1. E Quantitative data for IHF of C18-4 and TM4 cell samples for the detection of catenin. F Quantitative data for IHF of C18-4 and TM4 cell samples for the detection of Cx43. G Quantitative data for IHF of C18-4 and TM4 cell samples for the detection of Cx37. H Quantitative data for IHF of C18-4 and TM4 cell samples for the detection of JAM1.I Quantitative data for IHF of C18-4 and TM4 cell samples for the detection of Phldb2. (n=6 times). Data are presented as mean ± SEM. a, b, c, d indicate a significant difference among different treatments (p < 0.05).

Supplemental Table 1. Primary antibodies information.

## Declaration of interest

The authors declare that there is no conflict of interest that could be perceived as prejudicing the impartiality of the research reported.

## Funding

This study was supported by the National Natural Science Foundation of China http://dx.doi.org/10.13039/501100001809 (32070536 and 31772408), and High-level Personnel Scientific Research Fund of Qingdao Agricultural University http://dx.doi.org/10.13039/100012900 (6651117004 to QS).

## Ethics approval

All procedures involving live mice were performed in accordance with the NIH Guide for the Care and Use of Laboratory Animals and the protocols approved by the Qingdao Agricultural University Animal Care and Use Committee. ICR mice were used in this investigation.

## Data availability

Source data associated with Figs 3, 5, and 6 can be accessed through GEO: GSE137628 and GSE142618. All data are reported in the main text and supplementary materials and are available from the corresponding author upon request.

## Author contribution statement

Y Z conceived and designed the study. X H and P Z performed animal experiments and RNA-seq data analysis. C Z, and B X performed histological analyses and IHF and WB. S Y and Z G performed the cell experiments. W S and H Z did the data analysis and help with the manuscript preparation. YZ prepared the manuscript with input from co-authors. All authors read and approved the final manuscript.
