# Supplementary figures and images for: Muscarinic acetylcholine receptor M5 is involved in spermatogenesis through the modification of cell–cell junctions
Source: Reproduction. 2021 May 10;162(1):47–59. doi: 10.1530/REP-21-0079 (PMC8183636; doi:10.1530/REP-21-0079)

A

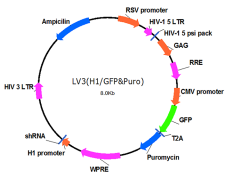

B

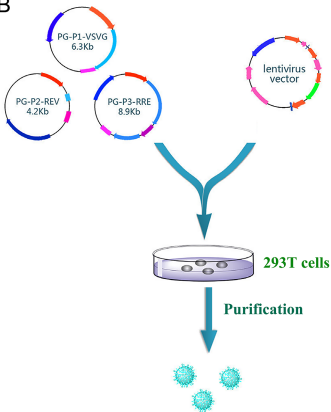

Supplement: Supplemental Fig. 1 Structure of the lentivirus vector used in this study and its production protocol for subsequent infection of cells and animals. [file supplementary_figure_1.pdf]

M5/Nuclei

M1/Nuclei

M3/Nuclei

1wk

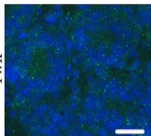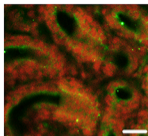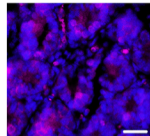

2wk

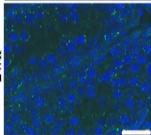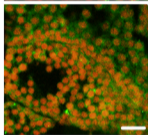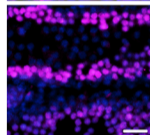

3wk

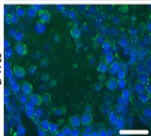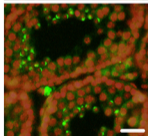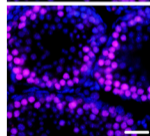

6wk

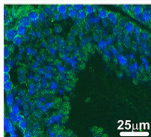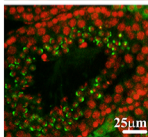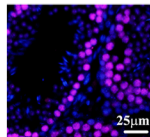

Supplement: Supplemental Fig. 2 Protein expression of muscarinic acetylcholine receptors (mAChRs) M1, M3, and M5 in mouse testes at the age of 1, 2, 3, and 6 weeks. Scale bar: 25 μm. [file supplementary_figure_2.pdf]

**A**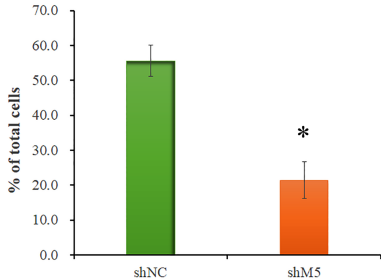**B**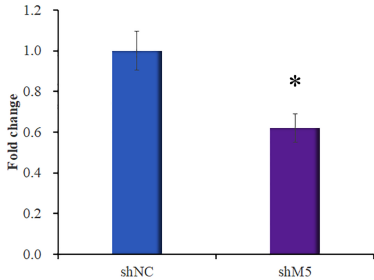

Supplement: Supplemental Fig. 3 Quantitative data for IHF and WB of mouse testis samples. A Quantitative data for IHF of mouse testis samples (for M5 detection). B Quantitative data for WB of mouse testis samples (for M5 detection). (n=6/group). Data are presented as mean ± SEM. a, b indicate a significant diff [file supplementary_figure_3.pdf]

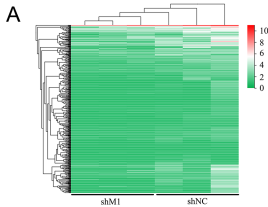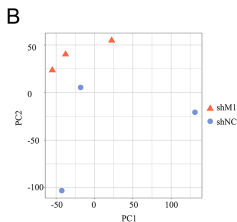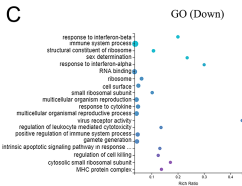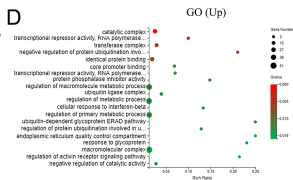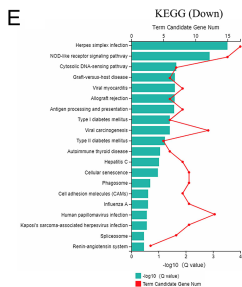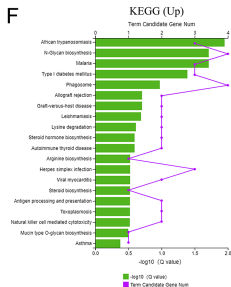

Supplement: Supplemental Fig. 4 Gene expression in mouse testes after M1 knockdown by short hairpin RNA (shRNA) in vivo. A Gene expression heatmap of mouse testis samples after M1 shRNA treatment for 10 days. B PCA analysis for the gene expression of mouse testes. C GO enrichment analysis of the genes decreased [file supplementary_figure_4.pdf]

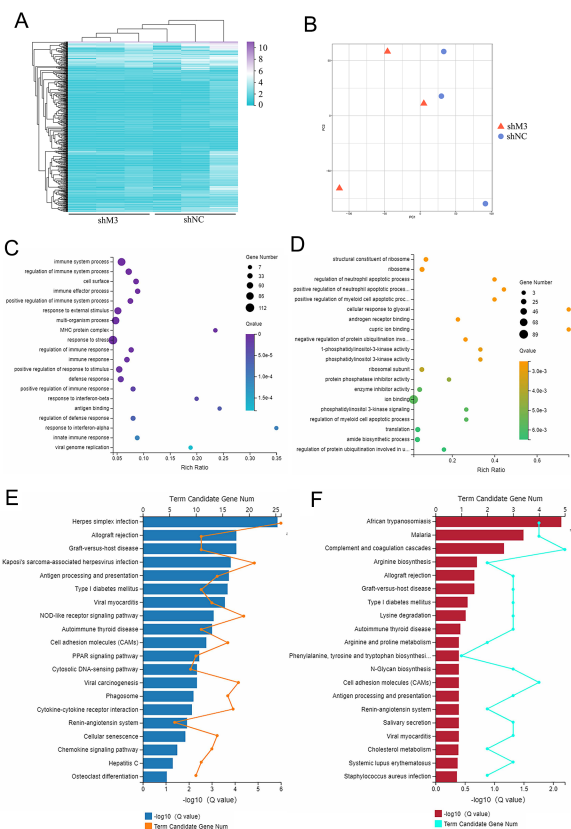

Supplement: Supplemental Fig. 5 Gene expression in mouse testes after M3 knockdown by short hairpin RNA (shRNA) in vivo. A Gene expression heatmap of mouse testis samples after M3 shRNA treatment for 10 days. B PCA analysis for the gene expression of mouse testes. C GO enrichment analysis of the genes decreased [file supplementary_figure_5.pdf]

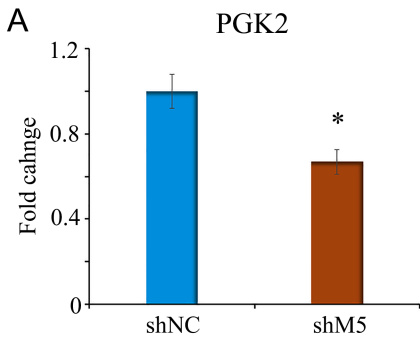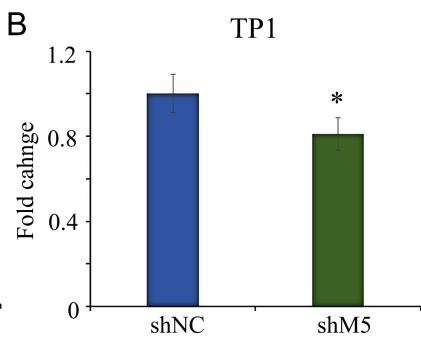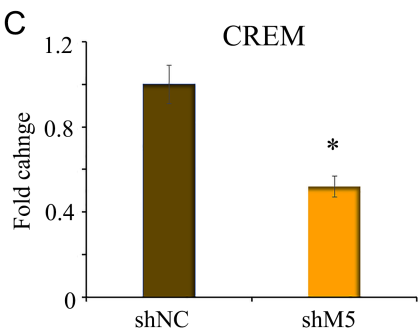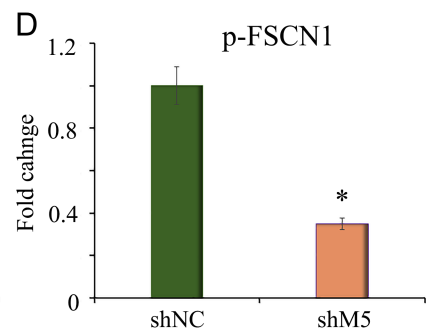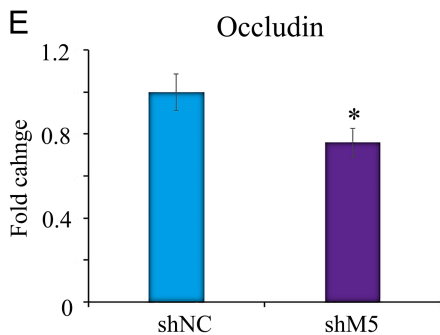

Supplement: Supplemental Fig. 6 Quantitative data for WB of mouse testis samples. A Quantitative data for WB of mouse testis samples for the detection of PGK2. B Quantitative data for WB of mouse testis samples for the detection of TP1. C Quantitative data for WB of mouse testis samples for the detection of CRE [file supplementary_figure_6.pdf]

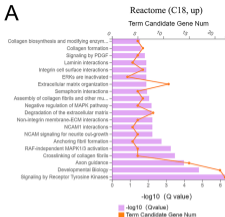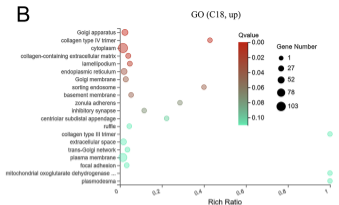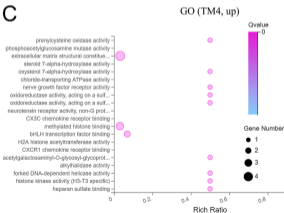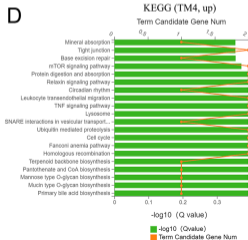

Supplement: Supplemental Fig. 7 The data summary of increased gene in C18-4 and TM4 cells after shM5 treatment in vitro. A Reactome enrichment analysis of the genes increased by shM5 treatment in C18-4 cells. B GO enrichment analysis of the genes increased by shM5 treatment in C18-4 cells. C Reactome enrichment [file supplementary_figure_7.pdf]

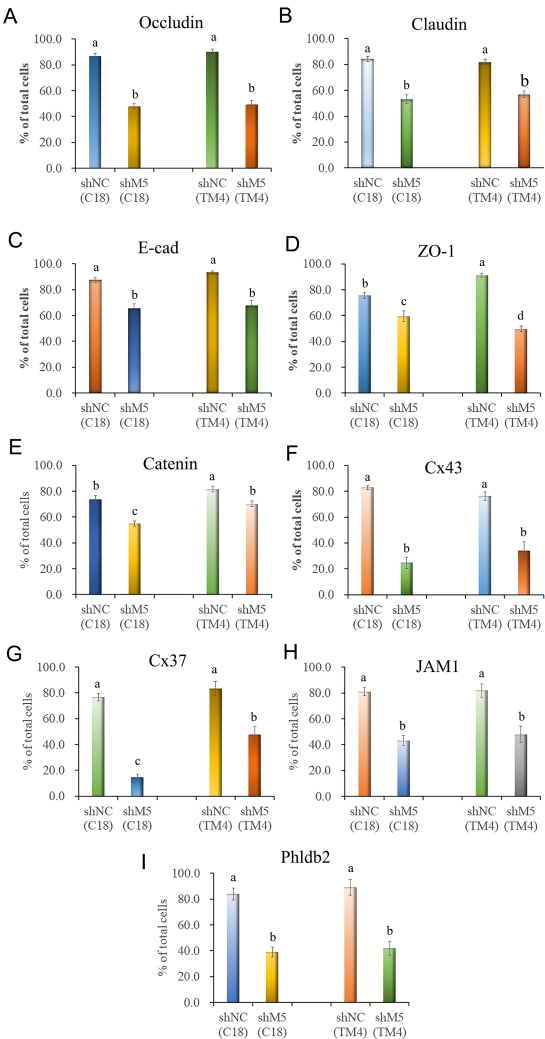

Supplement: Supplemental Fig. 8 Quantitative data for IHF of C18-4 and TM4 cell samples. A Quantitative data for IHF of C18-4 and TM4 cell samples for the detection of occludin. B Quantitative data for IHF of C18-4 and TM4 cell samples for the detection of claudin. C Quantitative data for IHF of C18-4 and TM4 c [file supplementary_figure_8.pdf]
