# Supplementary material for: Muscarinic acetylcholine receptor M5 is involved in spermatogenesis through the modification of cell–cell junctions
Source: Reproduction. 2021 May 10;162(1):47–59. doi: 10.1530/REP-21-0079 (PMC8183636; doi:10.1530/REP-21-0079)
Supplement: Supplemental Table 1. Primary antibodies information. [file supplementary_table_1.pdf]

**Table S1.** Primary antibody information

| Gene symbol | Name | Cat. # | Predicted size | Source (Animal) | Company |
|---|---|---|---|---|---|
| GAPDH | Glyceraldehyde-3-phosphate dehydrogenase | sc-48166 | 37kd | Goat (polyclonal) | Santa Cruz Biotechnology, Inc. |
| PHLDB2 | Pleckstrin Homology Like Domain Family B Member 2 | Ab234885 | 142kDa | Rabbit (polyclonal) | Abcam |
| M1 | Anti-Muscarinic Acetylcholine Receptor 1 | Ab-180636 | 51kDa | Rabbit | Abcam |
| M3 | Anti-Muscarinic Acetylcholine Receptor M3 | Ab-126168 | 66kDa | Rabbit | Abcam |
| M5 | Anti-Muscarinic Acetylcholine Receptor M5 | Ab-186830 | 60kDa | Rabbit | Abcam |
| SOX9 | SRY (sex-determining region Y)-box 9 protein | AB5535 | 65kDa | Rabbit (polyclonal) | Merck Millipore |
| TNP1(TP1) | Transition protein-1 | ab73135 | | Rabbit (polyclonal) | Abcam |
| p-FSCN1 | Fascin homolog 1 actin bundling protein (phospho S39) | bs-0772R | 55kd | Rabbit (polyclonal) | Beijing Biosynthesis Biotechnology CO. |
| CREM | cAMP response element modulator | D152356 | 39kd | Rabbit (polyclonal) | Sangon Biotech (Shanghai) Co., Ltd. |
| Cx37 | Connexin37 | bs-4067R | 37kDa | Rabbit (polyclonal) | Beijing Biosynthesis Biotechnology CO. |
| Cx43 | Connexin 43 | bs-0651R | 42kDa | Rabbit (polyclonal) | Beijing Biosynthesis Biotechnology CO. |
| JAM1 | Junction adhesion molecule 1 | bs-3651R | 30kDa | Rabbit (polyclonal) | Beijing Biosynthesis Biotechnology CO. |
| PGK2 | Anti-PGK2 rabbit polyclonal antibody | D121803 | 45kDa | Rabbit (polyclonal) | Sangon Biotech . |
| E-cadherin | Anti-E Cadherin | ab11512 | | rat | Abcam |
| Occludin | Rabbit Anti-Occludin antibody | bs-10011R | 59kDa | Rabbit (polyclonal) | Beijing Biosynthesis Biotechnology CO. |
| Claudin-11 | Rabbit Anti-Claudin 11/Oligodendrocyte Specific Protein antibody | bs-2183R | 22kDa | Rabbit (polyclonal) | Beijing Biosynthesis Biotechnology CO. |
| Catenin | Recombinant Anti-delta 1 Catenin/CAS antibody | Ab-92514 | 108kDa | Rabbit | Abcam |
| Zo-1 | ZO1 tight junction protein | Ab-190085 | 191kDa | Goat | Abcam |
